# The effectiveness of Dutch Cell Dogs in correctional facilities in the Netherlands: a study protocol of a quasi-experimental trial

**DOI:** 10.1186/s12888-018-1797-5

**Published:** 2018-07-05

**Authors:** Gerdien Schenk, Hanne M. Duindam, Hanneke E. Creemers, Machteld Hoeve, Geert Jan J. M. Stams, Jessica J. Asscher

**Affiliations:** 1 Research Institute of Child Development and Education, University of Amsterdam, Amsterdam, Netherlands; 2 Child and Adolescent Studies, Utrecht University, Utrecht, Netherlands

**Keywords:** Dutch cell dogs (DCD), Prison based animal Program’s (PAP), Treatment, Cortisol, Judicial care

## Abstract

**Background:**

Many former inmates recidivate, resulting in high costs for societies worldwide. Evidence based treatment practices may not work in prisons, due to detainees’ lacking motivation, impaired well-being, and an unsafe group environment. One attempt to improve social group climate and well-being is the use of Prison-based Animal Programs (PAP). Using a quasi-experimental design, the aim of the current study is to examine the effectiveness of one such PAP in the Netherlands: Dutch Cell Dogs (DCD).

**Methods/Design:**

Participants (*N* = 256) from 12 justice centers, including psychiatric, juvenile and adult facilities, will be recruited. Half of the sample (*n* = 128) will receive DCD training after voluntarily signing up (intervention group); The other half (*n* = 128) will be recruited to participate in the research and receive treatment-as-usual (TAU/Ccomparison group). Factors related to psychosocial functioning (e.g., self-esteem, empathy, self-control, life satisfaction, attention) and general therapeutic factors (i.e., therapeutic alliance, treatment motivation), expected to contribute to treatment success, will be assessed to measure the effectiveness of DCD. In addition, behavioral problems will be measured as well as recidivism rates. Questionnaires and neuropsychological tests will be employed to measure aforementioned outcome variables. Moreover, physiological data, based on heart rate and cortisol measures, will be collected to provide insight into the functioning of participants’ physiological stress response and to determine whether stress reduction occurs over time. Multimethod data collection will occur at pre-training (T1), at 1-month (halfway training/T2), at 2-months (end training/T3), and 6-months after the end of the training (follow up/T4).

**Discussion:**

This is the first study to examine the effectiveness of a widely implemented PAP in the Netherlands. Challenges associated with conducting the proposed study are typical for practice based research in correctional settings (e.g., a demanding workload of staff, lack of motivation to participate in research). Study results on the effects of a PAP will have an impact on inmates, justice centers, and municipalities across the Netherlands.

**Trial registration:**

Retrospectively registered. The Netherlands National Trial Register TC = 6894.

## Background

Recidivism after incarceration is high, with reconviction rates ranging from 20 to 59% within two years after imprisonment [1]. Although various treatments are available in correctional facilities to reduce the risk of recidivism, few such interventions have proven effective. In their goal to effectively reduce the risk of recidivism, therapeutic approaches in the last decade increasingly focus on general therapeutic factors that may help interventions to be effective. These general factors include treatment motivation, the therapeutic alliance, and the social climate in correctional facilities [2]. It is unclear, however, whether, and to what extent, a focus on these general factors contributes to positive treatment outcomes [3].

One method to make detainees more responsive and receptive to treatment is the use of Animal Assisted Interventions (AAI). AAIs have been associated with improved outcomes in four areas: autism spectrum symptoms, medical difficulties, emotional well-being, and behavioral problems [4]. Interaction with animals is believed to have beneficial effects in various ways. It is supposed to improve attachment relationships, provide the opportunity to be altruistic and caring, offer support in the form of ‘someone to talk to’, and may function as an opportunity to develop social capital [5]. Research on dog-owner interaction specifically also points at increased social interactions, as encounters with familiar and unfamiliar people become easier when accompanied by a dog [6]. In addition, Holbrook and colleagues stated that the attachment relationship with a dog makes people in general more open to improving and starting relationships with others [7]. Furthermore, interaction with dogs has been associated with physiological changes. Dotson and Hyatt showed that the company of a dog lowers blood pressure and diminishes stress [6]. Lower stress levels have been associated with a decrease in antisocial behavior [8]. Beetz and others showed a decreased heart rate in juveniles when interacting with a dog [9]. In sum, previous research suggests that interacting with animals has various positive consequences, which provides the framework for AAIs.

One form of AAI is the Prison-based Animal Program (PAP), in which animals are used to increase well-being and stimulate positive behavioral change of detained juveniles and adults. In the United States (US), animals most commonly used in PAPs are dogs. In this context, PAPs with various approaches and goals are often small-scale and serve a heterogeneous group of detainees. As a result, establishing effectiveness is complicated [5, 10, 11]. Nevertheless, the few studies that have been conducted in this area show positive results. Furst concluded that detainees experienced a connection with their training dogs after a short period of time [11]. Moreover, they felt responsible for and were empathetic towards their dogs and trusted the animals. Jasperson found that contact and collaboration among detainees and between detainees and group workers became easier in the presence of an animal, which improved group climate [12]. Fournier and colleagues demonstrated that the use of a PAP in the US resulted in increased self-control and less offenses [13]. The latter finding is consistent with findings by Lai, who showed a decrease in registered recidivism for detained juveniles who participated in a training program with asylum dogs [14]. Cooke and Farrington conducted a meta-analysis on PAPs in the US [10]. They included 10 studies that quantitatively evaluated PAPs, and found significant mean effects sizes for reductions in externalizing (i.e., recidivism, behavioral infractions, self-control) and internalizing (i.e., self-esteem, depression, self-efficacy, well-being) behavioral problems. In sum, the studies that have been conducted suggest that PAPs tend to have positive effects on detainees, and therefore may be an important contributor to successful rehabilitation.

Even though the results on the benefits of human-dog interaction and PAPs seem promising, findings have to be interpreted with caution. Many published evaluations of PAPs included small sample sizes without a control group. Some publications also had methodological limitations such as the use of instruments with unknown validity and reliability estimates [10, 15]. High-quality studies, conducted in a prison-based context, can provide insight into the functioning and effects of PAPs, and assist with the development of evidence-based practices. More research is urgently needed, because it has not kept up with the rise in popularity of PAPs worldwide [15].

To our knowledge, the study introduced in this protocol is the first to evaluate a PAP in a controlled manner, using a large sample size (*N* = 256) and including psychological, behavioral, and physiological outcome measures. It aims to examine the effectiveness of Dutch Cell Dogs (DCD), an 8-week training program in which detainees train asylum dogs. The DCD program has been carried out in the Netherlands since 2009, in various correctional facilities across the country. It targets both detained juveniles as well as adult offenders, including inmates with severe psychiatric problems. The overall objective of DCD is to create a win-win situation for two groups that have been placed outside society: detainees and asylum dogs. As with other PAPs, the assumption is that inmates – by training the behaviorally challenging dogs – acquire skills that are essential to mental health, such as self-esteem, empathy, social behavior, and self-control.

In the Netherlands, dogs sometimes are kept as a guardian dog, but in most cases they live in the houses of families as pets. Dogs are usually treated as family members and often a warm affectionate bond between the dog and family is established [16]. Based on AAI, human-dog interaction, and PAP research, DCD participation is hypothesized to improve general therapeutic factors (i.e., therapeutic alliance, treatment motivation) [6, 7], diminish anxiety and stress levels [9, 17], and reduce behavioral incidents in correctional settings and overall recidivism [14]. By decreasing anxiety and stress levels, detainees are believed to become less impulsive and experience more self-control [9]. These hypotheses are tested in a quasi-experimental study with assessments at pre-training (T1), one-month after the start of the training (halfway training/T2), two-months after the start of the training (end training/T3), and a follow up assessment six-months after the completion of the DCD training (follow up/T4). Research questions are:

1. To what extent is participation in the DCD training associated with an increase in psychosocial (i.e., self-confidence, empathy, self-control, self-reported stress, life satisfaction, social behavior) and physiological (i.e., reduction in basal cortisol levels and increase in heart rate variability) functioning in addition to general therapeutic factors (i.e., therapeutic alliance, treatment motivation)?
2. To what extent are potential changes in psychosocial and physiological functioning, as well as general therapeutic factors, associated with a higher treatment success ratio?
3. To what extent does the DCD training result in a decrease in internalizing (i.e., anxiety and depression) and externalizing (i.e., aggression and incidents) behavioral problems and recidivism?

## Methods/Design

### Study setting and eligibility criteria

This evaluation employs a quasi-experimental study design to determine the effects of a DCD training program on detainees. This study and its amendments have been approved by the Ethical Committee of the University of Amsterdam (2015-CDE-6363). If applicable, future amendments will be sent to the Ethical Committee for approval.

This study will be performed in the Netherlands, where 8019 adult and 426 juvenile detainees resided in respectively 24 general and 7 juvenile justice centers in 2016 [18]. Psychiatric justice centers also housed 1374 adult detainees in 2016. This type of center retains adults with psychiatric and/or developmental difficulties, who committed a serious offense and have a high recidivism risk [19]. Detainees can participate in this study when DCD is offered at their justice center between 2017 and 2018. Table 1 displays the correctional facilities that take part in the study, ordered by type of setting (juvenile, psychiatric adult, general adult).

**Table 1:** Participating justice centers, organized by type of facility

Type of justice center	Name of justice center
Juvenile justice center	Den Hey Acker, Breda De Hartelborgt, Spijkenisse De Hunnerberg, Nijmegen JJI Veenhuizen, Veenhuizen
Psychiatric justice center – adult population	De Rooyse Wissel, Oostrum De Woenselse Poort, Eindhoven Oostvaarderskliniek, Almere
Justice center – adult population	PI Zuyderbos, Heerhugowaard PI Achterhoek, Zutphen PI Almelo, Almelo PI Lelystad, Lelystad PI Nieuwersluis, Utrecht

The experimental group consists of participants who expressed motivation and are selected to take part in the DCD training. The same inclusion criteria that are used for DCD training participants are applied to the comparison group, which are 1) that detainees are physically able to participate in the training and strong enough to walk a dog, 2) that they are sufficiently sensitive in play and interaction with the dog, regardless of potential medication use, to ensure safety of the detainees and the asylum dogs; in order to prevent bite incidents and aggression, 3) duration: detainees remain in the correctional facility for at least two months after the start of the training. The comparison group is selected from the remaining detainees, who follow treatment-as-usual (TAU) in the justice centers, but cannot or do not currently want to partake in a DCD training.

A complicating factor for recruitment of participants for the study is the relatively low number of detainees per facility applying for the DCD program, ranging between six to ten detainees. DCD training groups consisting of six client-dog dyads are considered optimal; therefore, randomly assigning the potential participants to the experimental and comparison group would result in training groups that are not sufficiently filled. Due to these sampling difficulties, a quasi-experimental design is employed instead of the preferred Randomized Controlled Trial (RCT). We are dependent on the clinical staff in correctional facilities and DCD to recruit participants for the training group, as well as the comparison group. In both groups, changes in the program, for example drop out of detainees and/or asylum dogs will be carefully registered.

### Participant recruitment

DCD trainers inform the principal investigator (PI/first author) about new training dates, and provide contact information of staff dedicated to facilitate DCD in the correctional facilities and trainings locations. Thereafter, the PI establishes contact with the justice centers and sets up a meeting to explain the study’s purpose, procedure, and study participants’ rights. Correctional facilities are then invited to participate in the study. To recruit participants for the experimental group, the PI is present during the intake of DCD, where DCD informs potential participants what to expect of the training. At this time, the PI also informs the detainees in a short and comprehensible manner about the objectives of the study, what participating in the study entails, and about the societal and scientific importance of the study. To recruit participants for the comparison group, convenience sampling will be employed. Flyers will be distributed in the correctional facilities with information about the study and compensation offered for participation. In addition, the researchers will actively recruit participants by presenting the study to detainees in the correctional facilities. Participants are eligible for the comparison group when they meet the inclusion criteria of DCD as specified in the section on eligibility criteria. The staff at justice centers also play a key role in assisting with participants’ recruitment, as they are generally more known to (and trusted by) the detainees, and the first ones to provide more detail about DCD and the research. If the DCD participants come from a department that specializes in, for example, a specific type of offender (e.g., sexual offenders) or specific care offered (e.g., psychiatric care), the comparison group will be selected from the same department. Unfortunately, probability sampling methods cannot be employed for this study as a relatively low number of participants apply for DCD, thereby excluding random group assignment and a waiting list control group.

After a detainee has expressed interest in participating in the study to the staff of the correctional facility, he or she will be invited to a meeting with the researchers. The following topics will be discussed during this meeting: voluntary participation, guaranteed anonymity, actions taken to ensure confidentiality, contact information of PI in case questions come up, and compensation for study participation. Informed consent will be obtained, and participants receive a copy of the informed consent form. Study participants will receive a small stipend or gift, as determined by the correctional facilities, as an appropriate reward and motivator for taking part in the research. Examples of rewards are shower gel, phone cards, candy for youth participants, and prison store credit. Once enrolled in the research, the PI and research assistants will give their best efforts to ensure participants remain in the study upon completion. Examples of these efforts include: 1) establishing a successful working alliance with research participants, 2) scheduling assessment times in collaboration with participants, and 3) increasing rewards for research participation at follow-up.

### Sample size

Per four-month period, DCD aims to train four to six groups. This would result in 48 to 72 participants per four months. The objective is to include 128 DCD training participants and 128 comparison group participants. In approximately two to three years, this goal should be achieved. The goal is to sample 128 juvenile and 128 adult participants (each group containing 64 DCD and 64 comparison group participants) to allow for separate analyses that will provide insight into the workings of DCD for each subsample. This sample size was based on a priori power analysis that illustrated that a sample size of *N* = 128 would allow for a medium effect size (.25) to be detectable, given a power of .80 and an alpha of .05.

### Study condition

#### Experimental treatment: Dutch Cell Dogs training

The DCD training is a program in which detainees train asylum dogs for two months, twice a week, for two hours per day. The dogs are in the justice centers for the duration of the session and return to the asylum when the training for that day is over. The learning principles of operant conditioning form the basis of the training, in which only positive reinforcement with the help of a clicker and rewards for the dog are used. The aim of DCD is to stimulate the resocialization of both detainees and shelter dogs [20]. Detainees train shelter dogs so that they become more adoptable. In Dutch shelters, dogs generally only receive the basic care (i.e., food and walks) and do not receive additional (socialization) training. DCD intents to make previously ‘unadoptable’ dogs adoptable by providing a basic training with the help of detainees, resulting in a placement rate of approximately 95% [20].

Participation in the DCD training requires responsibility and dedication; therefore a detainee is eliminated from the training program if one session is missed without a valid reason. Likewise, when the asylum dog displays unsafe behavior to other dogs or people, i.e., falling out by barking, the detainee receives a yellow card. Two yellow cards make one red card, which again means elimination from the training. Finally, detainees are expelled when substance use is shown during regular institutional check-ups, and after expressing or causing severe forms of aggression and incidents. The training consists of four phases, described in Table 2. In addition to following the DCD training, participants in the experimental group may or may not follow treatment-as-usual (TAU) offered at the respective correctional facilities.

**Table 2:** Training content of the four phases

Training phase	Training content
One: The intake Three days prior to first training session	The DCD trainer explains principles underlying the training. Detainees are introduced to behavioral expectations during training; detainee chooses whether he/she wants to participate. If yes, the detainee is matched with an asylum dog (who has already undergone a behavioral test). The DCD trainer matches a shelter dog to a detainee based on observations during the intake, taking into consideration the safety of the dog and detainee at all times. The DCD trainer is not aware of the diagnoses and the offenses of the detainees. The dog-detainee matched is solely based on observations during the intake.
Two: The dog training: in theory and in practice Week 1–7	Training content is taught prior to each session. Detainees take notes in training diaries. Examples of training topics: teaching the dog desired behavior and how to respond to basic commands, taking care of the dog, and relaxation by playing. Detainees learn to recognize, interpret, and anticipate body language, emotions and behaviors of the dog. Extra attention is paid to learning how to understand and handle dog aggression.
Three: Graduation day. Week 8	The training ends with a celebratory ‘graduation’ day: detainees and their dogs demonstrate what they have learned to those interested (e.g., the group leaders of the detention center, family members, staff of the asylum centers). Detainees receive a certificate from the director of the correctional facility, in presence of their guests. They also receive a framed picture of ‘their’ dog and a t-shirt.
Four: Evaluation One month after training termination.	Detainees meet with DCD trainer to evaluate training experience. They are invited to provide feedback and various topics are discussed (i.e., anecdotes of training experience, the experience of saying goodbye to the dog). Detainees are also updated on the current living situation of the dog they trained (i.e., has the dog found a new home).

#### Comparison group: Treatment-as-usual

Participants in the comparison group either receive TAU at their respective justice centers or choose not to partake in therapy. TAU in justice centers in the Netherlands can include (but is not limited to) the following: Schema-Focused Cognitive Therapy, Multisystemic Therapy, Psychotherapy, Occupational Therapy, and/or the Liberman Addiction Module.

### Data collection

Data collection will be carried out by the PI and trained research assistants (i.e., students and recent graduates) at four separate time points: at pre-training (T1), one-month after the start of the training (T2), at the end of the training (T3), and a follow up assessment six months post-training completion (T4). Research assistants will be extensively trained prior to data collection. Table 3 displays the outcome measures that will be utilized to assess the effectiveness of DCD at different time points. Data will be collected by using various methods: questionnaires, interviews, neuropsychological tests, and physiological assessments of the autonomic nervous system. In addition, official judicial data will be requested from the Dutch Ministry of Justice in order to determine recidivism rates of study participants. Participants will also be requested to provide self-report data on offenses via an email survey.

**Table 3:** Variables’ instruments and sources

Outcome	Variable name	Instrument	Time of assessment	Variable type	Source
Demographic variables	Demographics	File analysis Questionnaire	T1 & T3	Moderator	Files, detainee
Psychosocial functioning	Self-esteem	RSES	T1, T2, T3, T4	Outcome	Detainee
	Self-control	BSCS, APSD-SR Impulse Control subscale, Emo go/no go task	T1, T2, T3, T4	Outcome	Detainee
	Self-reported stress levels	PSS	T1, T2, T3, T4	Outcome	Detainee
	Life satisfaction	SWLS	T1, T2, T3, T4	Outcome	Detainee
	Withdrawn behavior	ASR withdrawn subscale	T1, T2, T3, T4	Outcome	Detainee
	Attention	ASR/YSR attention subscale, Emo go/no go task	T1, T2, T3, T4	Outcome	Detainee
Emotional functioning	Empathy	BES	T1, T2, T3, T4	Outcome	Detainee
		Emo film clip	T1	Moderator	Detainee
	Emotional processing	Emo go/no go task	T1, T2, T3, T4	Outcome	Detainee
Physiological stress response	ANS-functioning	Heart rate variability	T1, T2, T3, T4	Moderator & outcome	Detainee
	Stress	Cortisol	T1, T3, T4	Moderator & outcome	Detainee
Therapeutic factors	Therapeutic alliance	PARA	T1, T2, T3, T4	Outcome	
	Treatment motivation	ATMQ	T1, T2, T3	Outcome	Detainee
	Responsivity to treatment	IFTE	T1, T3	Outcome	Staff
Training factors	Attachment relationship detainee - asylum dog	PBS	T2, T3	Outcome	Detainee intervention group
	Training appreciation	Structured interviews	T1, T2, T3, T4	Outcome	Detainee intervention group
Behavioral problems	Anxiety & depression	ASR/YSR anxious/depressed subscale	T1, T2, T3, T4	Outcome	Detainee
	Aggression	ASR aggression subscale	T1, T2, T3, T4	Outcome	Detainee
	Incidents	File analysis	T3	Outcome	Files
	Recidivism	Official judicial data, & email survey	T4	Outcome	Researcher & detainee
Personality functioning	Callous traits	ICU callous-unemotional traits subscale	T1, T3, T4	Moderator	Detainee
	Personality difficulties	PID-5-BF-NL	T1, T3, T4	Moderator & outcome	Detainee
Response style	Social Desirability	SDS	T1	Moderator	Detainee

Data collection will take place in a quiet room in the respective correctional facilities. One assessment will last for approximately 60 to 90 min. Participants will be presented with the questionnaires and neuropsychological tests on a computer screen that is placed approximately 50 cm in front of them. The questionnaires selected have been developed or successfully used in an adolescence and (young) adult population; therefore they are appropriate for the current study. The adolescent version of a questionnaire is provided to the participants in the juvenile justice centers, as specified in the next section. Additionally, reading assistance is offered to all participants at the start of the first assessment to ensure the comprehensibility of the questionnaires. In order to minimize potential strain caused by the research and to ensure reliability of research responses, participants will be closely monitored and offered to take breaks whenever deemed necessary. The assessment program will be run by a program called ‘Presentation’. Demographic information such as name, date of birth, ethnicity, offense type, recidivism risk, psychiatric diagnosis, treatment followed during training, living circumstances, school level, etc. will be obtained through file analysis (at post-intervention) and a self-report questionnaire.

### Outcome measures

#### Psychosocial functioning

The following indicators of psychosocial functioning will be assessed: self-esteem, self-control, (self-reported) stress levels, life satisfaction, withdrawn behavior, and attention ability. *Self-esteem* will be assessed by using the Dutch version of the Rosenberg’s Self Esteem Scale (RSES) [21, 22]. The scale consists of 10 items to be answered on a 4-point Likert scale ranging from 1 = totally disagree to 4 = totally agree.

*Self-control* will be assessed by using the Dutch Brief Self Control Scale (BSCS) [23, 24]. This instrument consists of 13 items to be answered on a 5-point Likert scale, ranging from 1 = strongly agree to 5 = strongly disagree. Moreover, the Impulse Control subscale of the Antisocial Process Screen Device – Self Report (APSD-SR) will be used to assess an additional aspect of self-control: the ability to control impulses [25]. This subscale consists of 5 items to be answered on a 3-point Likert scale, ranging from 1 = very untrue to 3 = very true. As an additional measure of participants’ ability to inhibit behaviors, the emotional go/no go task will be used. For the current study, the emotional go/no go task as described by Iria, Barbosa, and Paixao [26] and Schulz and colleagues [27] was adapted. Participants will be presented with a 48-picture set representing faces, each depicting one of four emotions (i.e., fear, anger, sadness, and neutral), during four blocks. In each block, participants will be asked to respond (‘go’) to a target emotion (sadness, anger, fear or neutral) and inhibit their response to the other emotions (‘no go’). For each block, a different target emotion will be randomly selected [27]. The majority of pictures presented in each round will be go-signals (75%), thereby creating a tendency for people to react (press ‘go’) to the presented pictures. Due to this inclination to respond, the number of commission errors (i.e., wrongly responding to a ‘no go’) and reaction time for commission errors give insight into participants’ behavioral inhibition [26, 27].

*Self-reported stress* level will be assessed by using a Dutch translation of the Perceived Stress Scale (PSS) [28]. This instrument consists of 10 items to be answered on a 5-point Likert scale, ranging from 1 = never to 5 = very often.

*Life satisfaction* will be assessed by using a Dutch translation of the Satisfaction With Life Scale (SWLS) [29]. This instrument consists of 5 items to be answered on a 7-point scale, ranging from 1 = completely disagree to 7 = completely agree.

*Withdrawn behavior* will be assessed by using the Dutch version of the Withdrawn Syndrome subscale of the Adult Self Report form (ASR) [30]. This subscale consists of 9 items to be answered on a 3-point Likert scale, ranging from 1 = not at all to 3 = often.

The ability to sustain *attention* will be assessed by using the Dutch version of the Attention subscale of the Adult and Youth Self Report forms (ASR/YSR) [30, 31]. The YSR will be used for participants in juvenile correctional facilities, whereas the ASR will be administered to adult participants. The subscale consists of 15 ASR items and an additional six items for the YSR version. Questions are to be answered on a 3-point Likert scale, ranging from 1 = not at all to 3 = often. In addition, the aforementioned emotional go/no go task will also give insight into the attentive ability of participants. Omission errors (missing a ‘go’ signal) will be calculated for each participant and provide a measure of attention [26, 27].

#### Emotional functioning

Empathy and emotional processing will be assessed as indicators of emotional functioning. *Empathy* will be measured by using two instruments. The Dutch version of the Basic Empathy Scale (BES) is used and consists of 20 items to be filled out on a 5-point Likert scale ranging from 1 = totally disagree to 5 = totally agree (Van Langen, Stams, Van Vught: Dutch validation of the basic empathy scale, unpublished). The BES provides a cognitive and affective empathy score [32]. In addition, empathy is measured by using empathy-eliciting film clips. In this task, participants will be shown a short, emotional, film-clip, depicting a sad scene, where a boy fails at selection training for a soccer tournament [33, 34]. To assess participants’ ability to identify the emotion of the boy, understand his emotion given the context, and share the boy’s experience of the emotion to some extent, follow up questions will be asked after the display of the short film clip. Participants are asked to identify the boy’s emotion by selecting one emotional cartoon out of six options (sadness, happiness, anger, fear, surprise, and a neutral face) and rate the intensity of this emotion. The same questions are asked of participants own emotions; responses are considered empathic when there is an overlap between the observed (of the boy) and experienced (of the participant) emotion [33, 34]. Heart rate is also measured during the display of the film clip, as this can help differentiate between sympathy and personal distress [33].

The emotional go/no go task mentioned above will be used as an additional measure to assess participants’ *emotional processing* ability to identify (negative) emotions. The differences in reaction time and the number of commission errors across the four emotional conditions (i.e., neutral, sad, angry, scared) provide an indication of participants’ ability to process and identify negative emotions (i.e., sad, angry, scared) [26, 27].

#### Physiological stress response

As an index of the functioning of the autonomic nervous system, *heart rate*, *heart rate variability*, and basal *cortisol levels* will be measured. Prior to the start of the assessment, participants will be connected to an ECG amplifier, developed by the Physiological Research Lab of the University of Amsterdam. Their heart rate will be continuously measured throughout the assessment. At set times, participants will be presented twice with a three-minutes, relaxing, film clip of aquatic sea life to measure resting heart rate [35]. Heart rate variability will be calculated of each participant based on their resting heart rate during the relaxing film clips. Additional information on participants’ physiological stress response, more specifically the functioning of their hypothalamus-pituitary-adrenal axis, will be collected in the form of non-invasive neuroendocrinological cortisol measurements. Saliva samples of the participants will be collected to assess their Cortisol Awaking Response (CAR) according to the CAR method, whereby participants will leave saliva on a salivette upon (1) awakening; (2) 30 min after awakening; (3) 60 min after awakening. Salivettes will be stored at − 25 degrees Celsius in a secured freezer at the University of Amsterdam. Cortisol concentrations will be measured with electrochemiluminescence immunoassay (ECLIA), a sensitive method for measuring cortisol with a lower detection limit of 0, 5 nmol/l [8].

#### Therapeutic setting factors

The *therapeutic alliance* with the group social worker or participants’ mentor is assessed with the Psychological Availability and Reliance on Adult instrument (PARA) [36]. This instrument consists of 19 items with a 4-point Likert scale, ranging from 1 = disagree to 4 = agree.

*Treatment motivation* is measured using the shortened version of the Adolescent Treatment Motivation Questionnaire (ATMQ) of Van der Helm [37]. This instrument consists of 11 items to be answered on a 3-point Likert scale, ranging from 1 = true to 3 = not true.

*Responsivity to treatment* is assessed using the Instrument for Forensic Treatment Evaluation (IFTE). This instrument is filled in by staff members of justice centers (i.e., group workers, therapists, mentors) and asks their assessment of 22 observational behavioral items that reflect detainees’ protective behaviors, problematic behaviors, and resocialization skills, thereby providing an indication for treatment responsivity. Items are rated on a 17-point scale with five general anchor points (i.e., none, rarely, sometimes, often, always) that describe the frequency of the behavior. Raters can also pick scores in between the anchor points to allow for the detection of behavioral changes over a short amount of time [38].

#### Training related factors

The *attachment relationship* between the detainee and the asylum dog is measured using the Pet Bonding Scale (PBS) (Angle: Utilization of the pet bonding scale to examine the relation between human/companion animals bond and self-esteem in pre-adolescence, unpublished). The PBS was slightly adjusted from its original version as the word ‘pet’ was replaced by ‘dog.’ The PBS consists of 25 items to be answered on a 3-point Likert scale, ranging from 1 = never to 3 = always.

Other measurements to study the training process include qualitative data obtained from the diaries of the detainees (i.e., they write a diary fragment each training session). Additionally, brief, structured interviews will take place at the end of each assessment at T1-T4 to assess participants’ *training experience*. This interview is specifically designed to detect the factors that make the training program unique and it provides important information that might be missed by the quantitative instruments.

#### Emotional and behavioral problems

*Internalizing problems*: anxiety and depression are measured using the ASR and YSR subscales anxious/depressed (ASR/YSR). These subscales consist of 18-items to be answered on a 3-point Likert scale, ranging from 0 = never to 2 = often/always [30, 31].

*Externalizing behavior problems*: aggression is measured using the aggressive behavior subscale of the ASR and YSR. This subscale consists of 15 (ASR) and 17 (YSR) items to be answered on a 3-point Likert scale, ranging from 0 = never to 2 = often/always [30, 31].

Incidents (i.e., fights, weapon possession, drug use) at the prison before and during the training are tracked by means of file analysis. This information is requested from the correctional facilities post-intervention (at T3).

Official judicial data will be requested by the Dutch Ministry of Justice in order to determine the *recidivism* rates. Additionally, detainees will be asked to provide self-report data on offenses via email at follow up.

#### Personality functioning

Participants’ *personality functioning* will be assessed by using the Dutch translation of the Personality Inventory for DSM-5 – Brief Form (PID-5-BF-NL) [39]. It assesses the Alternative Model of Personality Disorder (AMPD), as presented by the DSM-5, which includes five trait dimensions: Negative Affectivity (NA), Detachment (De), Antagonism (An), Disinhibition (Di), and Psychoticism (Ps) [40]. This instrument consists of 25 items, to be answered on a 4-point Likert scale, ranging from 0 = very false or often false to 3 = very true or often true.

In addition, participants’ *callous traits* will be assessed using the callous-unemotional traits subscale of the Inventory of Callous-Unemotional Traits (ICU) (Frick: The inventory of callous-unemotional traits, unpublished). This subscale consists of 24 items to be answered on a 4-point Likert scale, ranging from 0 = very false to 3 = always true.

#### Response style

To assess participants’ *response style* and their tendency to answer questions in a socially desirable manner, the Dutch translation of the Social Desirability Scale will be used (SDS) [41]. This instrument consists of 15 items to be answered with 1 = true or 2 = false.

### Data management

Upon receiving the written informed consent forms of detainees, an individual research number will be assigned to each participant. This number will be used on questionnaires and all other research materials (e.g., saliva salivette sets) to ensure anonymity and protect participants’ privacy. Participants’ identifying information (i.e., name, date of birth) will be stored in a password secured file that is only accessible to the PI and the research assistants of the project. Furthermore, all data will be stored in a secured cabinet in a locked room at the University of Amsterdam. The PI will oversee that data is stored and managed in a protected, de-identified, manner, adhering to the Dutch Personal Data Protection Act. Finally, study results will be reported anonymously.

### Data analyses

Before conducting the analyses, missing data will be addressed using multiple imputations [42]. The research questions will be answered using ANCOVA’s, with experimental condition as factor (DCD versus comparison) and the outcome variables (i.e., psychosocial and general therapeutic factors, physiological functioning, and behavioral problems) on post-test as dependent variables. Potential differences in outcome variables at pre-test between the experimental (DCD) and comparison group will be included as covariates in the analyses. In addition, we will examine whether there are systematic differences between the groups on factors expected to influence the outcome variables (e.g., other treatments received, participants’ general functioning in the facilities prior to the intervention). Potential differences will also be controlled for in the analyses. To determine whether the DCD training contributes to a difference in recidivism, Cox regression analyses will be conducted to determine whether the survival curve of detainees in de DCD training program is different from the comparison group. To determine whether there is a difference between experimental and control group in the number of offences committed, analysis of variance will be used. In these analyses, the following variables will be included as covariates: medication use, age, gender, race, severity/type of crime, substance abuse, prison sentence, and psychopathology.

## Discussion

The proposed research is the first study in the Netherlands to examine the effectiveness of DCD, a PAP, in terms of improving psychosocial functioning of detainees, reducing their emotional and behavioral problems and recidivism, and stimulating factors that may enhance detainees’ treatment success. Despite the growing popularity of PAPs worldwide, research into its effects on detainees has been scarce and often lacking in quality [15]. The current study provides a unique opportunity as it intends to evaluate a PAP on a large-scale, including a control group from the different correctional facilities where DCD is offered. Moreover, a multimethod approach, encompassing neuropsychological and physiological measurements, will be used to evaluate the training in a pre-post design with assessments at multiple time point, including a follow up at 6-months after the end of the training.

Despite the importance of shedding light on the functioning of PAPs, there are several challenges associated with conducting the current program evaluation of DCD. First of all, conducting research in correctional facilities is generally challenging: detainees depart or get replaced; high staff turnover and workload make staff not always sufficiently motivated for research. Moreover, the staff is generally not very familiar with research [43]. Detainees might not always be motivated to participate in research either, as they are frequently asked as a subject of study. However, clinical practice itself (i.e., DCD) has requested the current program evaluation and as their relationship with the correctional facilities is positive, the expectation is that effective collaboration between all involved parties will follow suit. This has also been the case in a previous, smaller-scale, research project between DCD, justice centers, and the University of Amsterdam. In addition, the university will commit to establish and maintain an effective working relationship with its community partners. For example, the PI will stay in frequent contact and offer support whenever necessary, as a good relationship between researchers and staff members is expected to be essential for conducting successful research in these ‘real life’ correctional settings [44]. Moreover, cooperation of management of correctional facilities is expected as they have expressed interest in the evaluation of DCD. If proven effective, evaluation results might open doors to additional funding opportunities that enables leadership of justice centers to offer more DCD trainings in their respective facilities.

An important limitation of the proposed study protocol, is that a quasi-experimental design is employed instead of the preferred RCT, thereby significantly increasing the risk of systematic biases. Unfortunately, conducting a RCT is impossible due to the low number of applicants for DCD in the correctional facilities. Potential differences between the intervention and comparison group at pre-test will be rigorously examined and controlled for by including them as covariates in the analyses. Even though a RCT cannot be employed, the proposed study is believed to be an improvement compared to previous research on PAPs because of its large sample size, control group, mixed method design, and inclusion of various outcome variables [15].

The proposed research project is expected to have an impact on a governmental level in the Netherlands, as from 2015 onward Dutch municipalities are responsible for all forms of youth services, including the implementation of child protection services and youth rehabilitation. Moreover, municipalities are responsible for the aftercare of juveniles and adults who re-enter in society after incarceration. Therefore, they are interested in innovative methods and interventions that may positively affect detainees’ psychosocial functioning, behavioral problems, and treatment outcomes, to ultimately help give people tools to successfully reintegrate after a period of incarceration. Thus, the proposed study provides a unique opportunity to examine the functioning of a PAP in correctional facilities across the Netherlands. If proven effective, it may be an innovative way to positively influence shelter dogs, detainees, and vicariously society at large.

## Abbreviations

AAI: Animal Assisted Intervention
AMPD: Alternative Model of Personality Disorder
An: Antagonism; ANS-functioning: Autonomic Nervous System-functioning
ANS-functioning: Autonomic Nervous System-functioning
ANCOVA: Analysis of Covariance
ASR: Adult Self Report form
ATMQ: Adolescent Treatment Motivation Questionnaire
BES: Basic Empathy Scale
BSCS: Brief Self Control Scale
CAR: Cortical Awakening Response
DCD: Dutch Cell Dogs
De: Detachment
Di: Disinhibition
ECG: Electrocardiogram
ECLIA: Electrochemiluminescence immunoassay
ICU: Inventory of Callous-Unemotional Traits
NA: Negative affectivity
PAP: Prison based Animal Program
PARA: Psychological Availability and Reliance on Adult
PBS: Pet Bonding Scale
PI: Principal Investigator
PID-5-BF-NL: Personality Inventory for DSM-5 – Brief Form – Nederlands (Dutch)
Ps: Psychoticism
PSS: Perceived Stress Scale
RCT: Randomized Controlled Trial
RSES: Rosenberg Self Esteem Scale
SWLS: Satisfaction With Life Scale
TAU: Treatment-as-usual
US: United States
YSR: Youth Self Report form

## References

[1] Fazel S (2015). Wolf a. A systematic review of criminal recidivism rates worldwide: current difficulties and recommendations for best practice. PLoS One.

[2] Wampold B (2001). The great psychotherapy debate: models, methods, and findings.

[3] van Yperen T, van der Steege M, Addink A, Boendermaker L. Algemeen en specifiek werkzame factoren in de jeugdzorg. Stand van de discussie. In: Rapport Nederlands Jeugdinstituut. 2010. https://www.nji.nl/nl/Download-NJi/Publicatie-NJi/Rapport-AlgemeenWerkzameFactoren.pdf. Accessed 6 Nov 2017.

[4] Nimer J, Lundahl B (2007). Animal-assisted therapy: a meta-analysis. Anthrozoös.

[5] Mercer J, Gibson K, Clayton D (2015). The therapeutic potential of a prison-based animal programme in the UK. J Forensic Pr.

[6] Dotson MJ, Hyatt EM (2008). Understanding dog–human companionship. J Bus Res.

[7] Holbrook MB, Stephens DL, Day E, Holbrook SM, Strazar G. A collective stereographic photo essay on key aspects of animal companionship: the truth about dogs and cats. Acad Market Sci Rev. 2001;1:1–17.

[8] Platje E, Vermeiren R, Raine A, Doreleijers TA, Keijsers L, Branje S, Popma A, van Lier P, Koot H, Meeus W (2008). A longitudinal biosocial study of cortisol and peer influence on the development of adolescent antisocial behavior. Psychoneuroendocr.

[9] Beetz A, Uvnas-Moberg K, Julius H, Kotrschal K (2012). Psychosocial and psychophysiological effects of human-animal interactions: the possible role of oxytocin. Front Psychol.

[10] Cooke BJ, Farrington DP (2016). The effectiveness of dog-training programs in prison: a systematic review and meta-analysis of the literature. Pris J.

[11] Furst G (2006). Prison-based animal programs: a national survey. Pris J.

[12] Jasperson RA (2010). Animal-assisted therapy with female inmates with mental illness: a case example from a pilot program. J Offender Rehabil.

[13] Fournier AK, Geller ES, Fortney EV (2007). Human-animal interaction in a prison setting: impact on criminal behavior, treatment progress, and social skills. Behav Soc Sci.

[14] Lai J. Pet facilitated therapy in correctional institutions In: Women Offender Programs and Issues. 1998. Correctional Services of Canada by Office of the Deputy Commissioner for women. http://www.csc-scc.gc.ca/publications/fsw/pet/pet-eng.shtml. Accessed 6 Nov 2017.

[15] Mulcahy C (2013). McLaughlin D. Is the tail wagging the dog? A review of the evidence for prison animal programs. Aust Psychol.

[16] Endenburg N, de Jonge FH, van de Bos R (2005). The death of a companion animal and human bereavement. The human-animal relationship. Forever and a day.

[17] O'haire M (2010). Companion animals and human health: benefits, challenges, and the road ahead. J Vet Beh: Clin Appl Res.

[18] de Looff J, van de Haar M, Gemmert N, Valstar H. Gevangeniswezen 2012-2016 & Justitiële Jeugdinrichtingen 2012-2016. In: DJI in getal 2012–2016. 2017. https://www.dji.nl/binaries/DJI%20in%20getal%202012-2016_tcm41-271319.pdf. Accessed 6 May 2018.

[19] Dienst Justitiële Inrichtingen. Tbs inzichtelijk. 2017. https://www.dji.nl/binaries/infographic%20tbs_2017_tcm41-271323.pdf. Accessed 6 May 2018.

[20] Wiegerinck H, Buij B. Dutch cell dogs Jaarverslag 2016. http://dutchcelldogs.nl/2016/wp-content/uploads/2017/11/DCD_jaarverslag2016.pdf. Accessed 6 May 2018.

[21] Rosenberg M (1979). Conceiving the self.

[22] Franck E, De Raedt R, Barbez C, Rosseel Y (2008). Psychometric properties of the Dutch Rosenberg self-esteem scale. Psychol Belg.

[23] Tangney JP, Baumeister RF, Boone AL (2004). High self-control predicts good adjustment, less pathology, better grades, and interpersonal success. J Pers.

[24] Finkenauer C, Engels R, Baumeister R (2005). Parenting behaviour and adolescent behavioural and emotional problems: the role of self-control. Int J Behav Dev.

[25] Frick PJ, Hare RD (2001). The antisocial process screening device.

[26] Iria C, Barbosa F, Paixão R (2012). The identification of negative emotions through a go/no-go task. Eur Psychol.

[27] Schulz KP, Fan J, Magidina O, Marks DJ, Hahn B, Halperin JM (2007). Does the emotional go/no-go task really measure behavioral inhibition? Convergence with measures on a non-emotional analog. Arch Clin Neuropsych.

[28] Cohen S, Kamarck T, Mermelstein R (1983). A global measure of perceived stress. J Health Soc Behav.

[29] Diener E, Emmons RA, Larsen RJ, Griffin S (1985). The satisfaction with life scale. J Pers Assess.

[30] Achenbach TM, Rescorla LA (2003). Manual for the ASEBA adult forms & profiles.

[31] Achenbach TM (1991). Manual for the youth self-report and 1991 profile.

[32] Jolliffe D, Farrington DP (2006). Development and validation of the basic empathy scale. J Adolesc.

[33] de Wied M, van Boxtel A, Matthys W, Meeus W (2012). Verbal, facial and autonomic responses to empathy-eliciting film clips by disruptive male adolescents with high versus low callous-unemotional traits. J Abnorm Child Psychol.

[34] Van der Graaff J, Meeus W, de Wied M, van Boxtel A, van Lier PA, Koot HM, Branje S (2016). Motor, affective and cognitive empathy in adolescence: interrelations between facial electromyography and self-reported trait and state measures. Cognition Emotion.

[35] Piferi RL, Kline KA, Younger J, Lawler KA (2000). An alternative approach for achieving cardiovascular baseline: viewing an aquatic video. Int J Psychophysiol.

[36] Schuengel C, Zegers M. Psychological availability and reliance on adult manual. Amsterdam: Free University Amsterdam; 2003.

[37] Van der Helm G, Wissink I, De Jongh T, Stams G (2013). Measuring treatment motivation in secure juvenile facilities. Int J Offender Ther Comp Criminol.

[38] Schuringa E, Heininga VE, Spreen M, Bogaerts S (2016). Concurrent and predictive validity of the instrument for forensic treatment evaluation: from risk assessment to routine, multidisciplinary treatment evaluation. Int J Offender Ther Comp Criminol.

[39] van der Heijden P, Ingenhoven T, Berghuis H, Rossi G (2014). Nederlandse bewerking van this personality inventory for DSM-5- brief form (PID-5-BF) - adult.

[40] American Psychiatric Association. Diagnostic and statistical manual of mental disorders (DSM-5®). Arlington: American psychiatric pub; 2013.

[41] Crowne DP, Marlowe D (1960). A new scale of social desirability independent of psychopathology. J Consult Psychol.

[42] Graham JW (2009). Missing data analysis: marking it work in the real world. Annu Rev Psychol.

[43] Simons I, Mulder E, Rigter H, Breuk R, van der Vaart W, Vermeiren R (2016). Family-centered Care in Juvenile Justice Institutions: a mixed methods study protocol. JMIR Res Protoc.

[44] Israel BA, Schurman SJ, Hugentobler MK (1992). Conducting action research: relationships between organization members and researchers. J Appl Behav Sci.

